# Evidence of a Bacterial Receptor for Lysozyme: Binding of Lysozyme to the Anti-σ Factor RsiV Controls Activation of the ECF σ Factor σ^V^

**DOI:** 10.1371/journal.pgen.1004643

**Published:** 2014-10-02

**Authors:** Jessica L. Hastie, Kyle B. Williams, Carolina Sepúlveda, Jon C. Houtman, Katrina T. Forest, Craig D. Ellermeier

**Affiliations:** 1 Department of Microbiology, Carver College of Medicine, University of Iowa, Iowa City, Iowa, United States of America; 2 Department of Bacteriology, University of Wisconsin Madison, Madison, Wisconsin, United States of America; University of California, San Francisco, United States of America

## Abstract

σ factors endow RNA polymerase with promoter specificity in bacteria. Extra-Cytoplasmic Function (ECF) σ factors represent the largest and most diverse family of σ factors. Most ECF σ factors must be activated in response to an external signal. One mechanism of activation is the stepwise proteolytic destruction of an anti-σ factor via Regulated Intramembrane Proteolysis (RIP). In most cases, the site-1 protease required to initiate the RIP process directly senses the signal. Here we report a new mechanism in which the anti-σ factor rather than the site-1 protease is the sensor. We provide evidence suggesting that the anti-σ factor RsiV is the bacterial receptor for the innate immune defense enzyme, lysozyme. The site-1 cleavage site is similar to the recognition site of signal peptidase and cleavage at this site is required for σ^V^ activation in *Bacillus subtilis*. We reconstitute site-1 cleavage *in vitro* and demonstrate that it requires both signal peptidase and lysozyme. We demonstrate that the anti-σ factor RsiV directly binds to lysozyme and muramidase activity is not required for σ^V^ activation. We propose a model in which the binding of lysozyme to RsiV activates RsiV for signal peptidase cleavage at site-1, initiating proteolytic destruction of RsiV and activation of σ^V^. This suggests a novel mechanism in which conformational change in a substrate controls the cleavage susceptibility for signal peptidase. Thus, unlike other ECF σ factors which require regulated intramembrane proteolysis for activation, the sensor for σ^V^ activation is not the site-1 protease but the anti-σ factor.

## Author Summary

All cells sense and respond to changes in their environments by transmitting information across the membrane. In bacteria, σ factors provide promoter specificity to RNA polymerase. Bacteria encode Extra-Cytoplasmic Function (ECF) σ factors, which often respond to extracellular signals. Activation of some ECF σ factors is controlled by stepwise proteolytic destruction of an anti-σ factor which is initiated by a site-1 protease. In most cases, the site-1 protease required to initiate the RIP process is thought to be the signal sensor. Here we report that the anti-σ factor RsiV, and not the site-1 protease, is the sensor for σ^V^ activation. Activation of the ECF σ factor σ^V^ is induced by lysozyme, an innate immune defense enzyme. We identify the site-1 protease as signal peptidase, which is required for general protein secretion. The anti-σ factor RsiV directly binds lysozyme. Binding of lysozyme to RsiV allows signal peptidase to cleave RsiV at site-1 and this leads to activation of σ^V^. Thus, the anti-σ factor functions as a bacterial receptor for lysozyme. RsiV homologs from *C. difficile* and *E. faecalis* also bind lysozyme, suggesting they may utilize this receptor-ligand mechanism to control activation of σ^V^ to induce lysozyme resistance.

## Introduction

Cells respond to changes in their environments using signal transduction systems, which transmit information from outside the cell across the membrane to effect transcriptional responses. Regulated Intramembrane Proteolysis (RIP) is one mechanism by which cells sense and respond to changes in the environment. The RIP signal transduction system was first described as the mechanism for controlling cholesterol biosynthesis in mammals [1]. In bacteria, RIP processes regulate the activity of several alternative σ factors including multiple Extra Cytoplasmic Function (ECF) σ factors. Most RIP signal transduction systems involve sequential cleavages of a membrane-tethered protein. Following site-1 cleavage by an initial protease, a second protease cleaves the substrate within the membrane at site-2. In most cases the rate-limiting step for activation of the signal transduction system is the cleavage of the substrate at site-1 [2]. Here we describe the role of RIP in regulating the activity of the *B. subtilis* ECF σ factor σ^V^ in response to lysozyme.

In bacteria, σ factors combine with RNA polymerase to recognize specific promoter sequences and transcribe mRNA. ECF σ factors represent a large and diverse family of important signal transduction systems in bacteria [3]. RIP regulates the activity of several alternative σ factors including multiple ECF σ factors in the subfamily ECF01 [2], . In *Escherichia coli*, activation of the ECF σ factor σ^E^ is initiated by site-1 cleavage of the anti-σ factor RseA when unfolded outer membrane β-barrel proteins bind to and activate the site-1 protease DegS [4]–[7]. In *Bacillus subtilis* activation of the ECF σ factor σ^W^ is thought to be controlled by activation of the site-1 protease PrsW, since mutants of PrsW were isolated which resulted in constitutive cleavage of the anti-σ factor RsiW even in the absence of stress [8]. In each of these cases the site-1 protease is thought to sense the signal required for activation of these ECF σ factors.

The *B. subtilis* ECF σ factor, σ^V^, belongs to the ECF30 subfamily of ECF σ factors, members of which are primarily found in firmicutes (low GC Gram-positive bacteria) [3]. A subset of the ECF30 homologs are controlled by anti-σ factors homologous to RsiV. σ^V^ is activated in response to lysozyme but not to other cell envelope stresses [9]–[11]. Lysozyme is an essential component of the host innate immune system which fights bacterial infection by cleaving cell wall saccharides and σ^V^ induces resistance to lysozyme [11]–[13]. We recently demonstrated that activation of σ^V^ requires the proteolytic destruction of the membrane tethered anti-σ factor, RsiV, in a RIP dependent mechanism [14]. This degradation requires the site-2 protease RasP [14]. RasP cleavage results in free σ^V^ which can complex with RNA polymerase and transcribe genes required for lysozyme resistance [9], [10]. Here we present evidence that the site-1 protease required for RsiV degradation is none other than signal peptidase. Signal peptidases are an essential component of the cellular secretion apparatus and are conserved from bacteria to eukaryotes. The activity of signal peptidases is not known to be regulated in response to environmental signals. We propose a model in which the sensor for cell envelope stress is the anti-σ factor RsiV. Our data indicate that RsiV is the direct receptor for lysozyme. Thus the anti-σ factor, and not the site-1 protease, is the sensor for lysozyme.

## Results

### Identification of the Site-1 Cleavage Site

**Figure 1:**
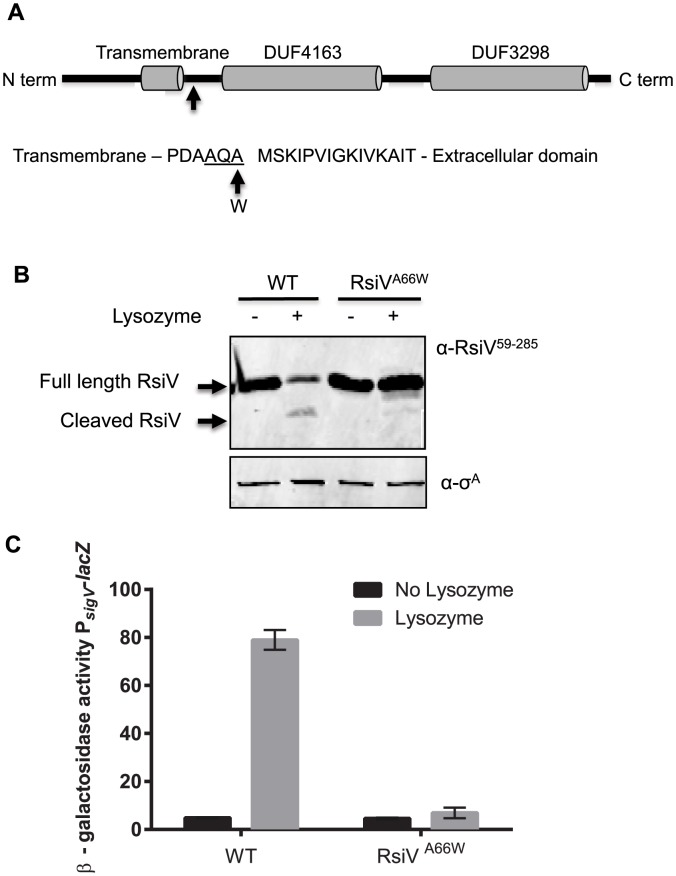
Site-1 cleavage of RsiV is required for σ^V^ activation. **A.** Model showing location of site-1 cleavage RsiV. Cells producing RsiV-6×His, which is under control of the IPTG inducible promoter P*_hyper-spank_* (P*_hs_*) (P*_hs_-rsiV-6×his* Δ*sigVrsiV::kan;* JLH548) were protoplasted with HEW lysozyme. RsiV-6×His was purified and the site-1 cleavage site was identified by N-terminal sequencing. The arrow denotes the amino acid residue 66 which was mutated to tryptophan. The transmembrane domain (AA 37–59) is based on the prediction of TMHMM [18]. The DUF domains, DUF4163 (AA 79–162) and DUF3298 (AA 188–270), are based on BLAST analysis [77] and the Pfam database [78]. The site-1 cleavage site between AA 66 and 67 is designated by an arrow. The sequence surrounding the site-1 cleavage site is shown the signal peptide cleavage site is underlined and the arrow and W denote the A66W mutation used to block site-1 cleavage. **B.** RsiV^A66W^ is not degraded in the presence of lysozyme. *B. subtilis* strains Δ*sigVrsiV::kan* P*_hs_*-*rsiV^+^* (JLH402) and Δ*sigVrsiV::kan* P*_hs_*-*rsiV^A66W^* (JLH623) were grown in LB+1 mM IPTG to an OD_600_ of 0.8. Samples were either treated with 2 µg/ml lysozyme (+) or untreated (−) and incubated for 10 minutes 37°C. Immunoblot was probed with anti-RsiV^59–285^ antibodies. **C.** RsiV^A66W^ inhibits σ^V^ activation. *B. subtilis* strains P*_sigV_-lacZ* (CDE1546) and P*_sigV_-lacZ rsiV^A66W^* (CDE2379) were grown overnight at 30°C and spotted onto LB and LB+HEW lysozyme (10 µg/ml) plates. Plates were incubated at 37°C for 6 hours and β-galactosidase activity was determined.

We previously demonstrated that activation of σ^V^ required the proteolytic destruction of the anti-σ factor RsiV in a RIP dependent mechanism [14]. Upon exposure to lysozyme we could detect what appeared to be the cleaved extracellular domain of RsiV [14] (Figure 1). This suggested that the extracellular domain is removed by an unknown protease that cleaves RsiV at site-1 after treatment with lysozyme. To determine the location of the site-1 cleavage, we constructed a *B. subtilis* strain producing a C-terminal 6×His tagged version of RsiV (RsiV^6×His^). Protoplasts of this strain were then generated using lysozyme. Following this treatment, we were able to purify the RsiV^6×His^ from the supernatant of these cells using nickel affinity resin (Figure S1). The sequence of the first 8 amino acids of the partially purified cleaved RsiV^6×His^ was determined by Edman degradation [15]. The N-terminal sequence of the cleaved RsiV domain was (MSKIPVIG) which indicates RsiV is cleaved between A66 and M67 (Figure S1 and Figure 1A).

### Disruption of Site-1 Cleavage Blocks RsiV Degradation and σ^V^ Activation

Analysis of the RsiV site-1 cleavage site revealed a canonical AXA motif suggestive of a signal peptide cleavage site. In fact subsequent analysis of RsiV *in silico* using SignalP [16] revealed a putative signal peptidase cleavage site between amino acids 66 and 67 of RsiV (Figure S2A). Furthermore an alignment of 185 RsiV homologs using Multiple Em for Motif Elicitation (MEME) a tool for identifying motifs in related sequences [17], reveals a highly conserved AXA motif just after their predicted transmembrane segment [18] (Figure S2B). In addition, analysis of *C. difficile*, *E. faecalis* and *B. subtilis* RsiV homologs using SignalP revealed the presence of a predicted signal peptidase cleavage site in each of those proteins (Figure S2A).

*B. subtilis* PY79 encodes five type 1 signal peptidases [19], [20]. SipS and SipT are the two major signal peptidases in *B. subtilis* and are redundant, but cells lacking both SipS and SipT are not viable [20]. We constructed *sipS* and *sipT* mutant strains and tested RsiV degradation and σ^V^ activation. We found that RsiV was still degraded in the absence of either SipS or SipT (Figure S3). We attempted to construct a strain to deplete signal peptidase however we were unsuccessful. Thus, to determine if disruption of cleavage at this site was sufficient to block RsiV degradation and σ^V^ activation, the alanine codon at position 66 was mutated to a tryptophan codon and tested for an effect on HEW lysozyme induced degradation. We found that while wild-type RsiV was rapidly degraded, the degradation of the RsiV^A66W^ mutant protein was blocked even in the presence of lysozyme (Figure 1B). This suggests that the RsiV^A66W^ mutant is unable to be cleaved at site-1. We then tested the effect of RsiV^A66W^ mutant protein on activation of σ^V^ in response to lysozyme by measuring expression of the P*_sigV_*-*lacZ* reporter. In cells producing wild type RsiV, expression of P*_sigV_-lacZ* was induced 16-fold in the presence of lysozyme (Figure 1C). In contrast, in strains producing RsiV^A66W^ there was no observable lysozyme induction of P*_sigV_-lacZ* expression. This indicates that the RsiV^A66W^ protein is resistant to site-1 cleavage, and thus, blocks activation of σ^V^ in response to lysozyme by inhibiting RsiV degradation. In addition, a strain producing RsiV^A66W^ is more susceptible to lysozyme than WT RsiV, due to an inability to degrade RsiV and activate σ^V^ (Table 1). Taken together this suggests that cleavage at site-1 is required for σ^V^ activation.

**Table 1:** 

Lysozyme Sensitivity Assay	WT	*sigVrsiV::kan*	*rsiV* ^A66W^
Lysozyme MIC	50 µg/ml	25 µg/ml	25 µg/ml
Zone of clearing lysozyme disk (10 mg/ml)	8 mm	11 mm	11 mm

### In the Presence of Lysozyme SipS Is Sufficient for Site-1 Cleavage of RsiV *In Vitro*

**Figure 2:**
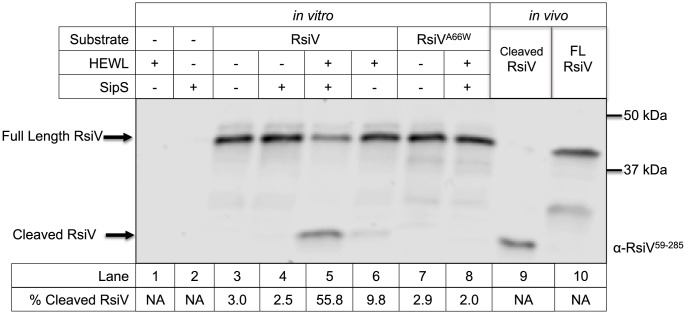
*In vitro* reconstitution of site-1 cleavage requires signal peptidase and lysozyme. Lane 1 contains 0.3 mg/mL purified HEW lysozyme; Lanes 2–7 contain cell free transcription/translation products derived using plasmids containing the gene(s) noted above the gel. Lane 2 SipS alone, Lane 3 RsiV alone; Lane 4 SipS+RsiV mixed at a molar ratio of 1∶3; Lane 5 SipS+RsiV mixed at a molar ratio of 1∶3+0.3 mg/mL HEW lysozyme; Lane 6 RsiV+0.3 mg/mL HEW lysozyme; Lane 7 RsiV^A66W^ alone; Lane 8 RsiV^A66W^+SipS mixed at a molar ratio of 1∶3+0.3 mg/ml HEW lysozyme. Lane 9 6×His-RsiV^59–285^ purified from *E. coli*; Lane 10 contains Full Length 3×Flag-CBP-RsiV prepared as extract from *B. subtilis* strain *amyE*::P*_hs_-3×flag-CBP-rsiV* Δ*sigVrsiV::kan* (JLH708) grown to OD_600_ 0.8. Arrows denote location of proteins on the gel FL-RsiV (3×Flag-CBP-RsiV-6×His) and Cleaved-RsiV (c-terminal). The gel was immunoblotted using anti-RsiV^59–285^ antibodies. The % Cleaved RsiV (Cleaved-RsiV/Total RsiV) was calculated by quantifying the intensities of the Full length RsiV and Cleaved RsiV bands using Li-Cor Image Studio [79].

Our data indicate that RsiV is cleaved at a putative signal peptidase cleavage site. Since signal peptidase activity is essential in *B. subtilis* [20] we sought to determine if signal peptidases were directly responsible for cleavage of the anti-σ factor RsiV *in vitro*. The signal peptidase SipS and RsiV both contain transmembrane domains and we hypothesized that these may be important for proper control of RsiV degradation. Thus, we produced SipS and RsiV *in vitro* using a cell free *in vitro* transcription/translation system. This method has been used successfully to test the ability of other membrane proteases to cleave their substrates [21]. Briefly, mRNA of *sipS* and *rsiV* was produced using SP6 RNAP as previously described [21]. The resulting mRNA served as a translation template using wheat germ extract as a source of ribosomes. Wheat germ extract contains sufficient endogenous lipids that at least some functional membrane proteins can be produced without addition of liposomes [21]. Both SipS and RsiV were produced in sufficient quantities that they could be visualized by Coomassie staining and they are present mostly in the insoluble pellet (i.e. lipid-containing) fraction of the *in vitro* translation reactions (Figure S4). We combined the SipS and RsiV reactions at 1∶3 molar ratios with or without the presence of HEW lysozyme and incubated the reactions at 37°C for 6 hours. Using anti-RsiV^59–285^ antibodies we were unable to detect the presence of any cleavage products of 3×Flag-CBP-RsiV when it was incubated alone or in the presence of SipS (Figure 2). When 3×Flag-CBP-RsiV was incubated with SipS in the presence of HEW lysozyme we observed the production of a cleavage product and a decrease in the amount of full length 3×Flag-CBP-RsiV (Figure 2). We observed minimal cleavage when 3×Flag-CBP-RsiV was incubated in the presence of HEW lysozyme, likely due to the presence of an eukaryotic signal peptidase in the wheat germ, which also recognizes an AXA motif [22], [23] (Figure 2). The cleaved product *in vitro* was the same size as the cleaved product produced from RsiV^6×His^ *in vivo* (Figure 2A).

We sought to determine if the A66W substitution in RsiV would block site-1 cleavage *in vitro* as it did *in vivo*. As seen in Figure 2 we observed only full length 3×Flag-CBP-RsiV^A66W^ when 3×Flag-CBP-RsiV^A66W^ was incubated with both SipS and HEW lysozyme (Figure 2). This suggests, in agreement with our *in vivo* results, that altering the signal peptidase recognition site in RsiV blocks site-1 cleavage *in vitro*. These data suggest that SipS is able to directly cleave RsiV and importantly this cleavage only occurs in the presence of HEW lysozyme.

### RsiV Binds HEW Lysozyme

**Figure 3:**
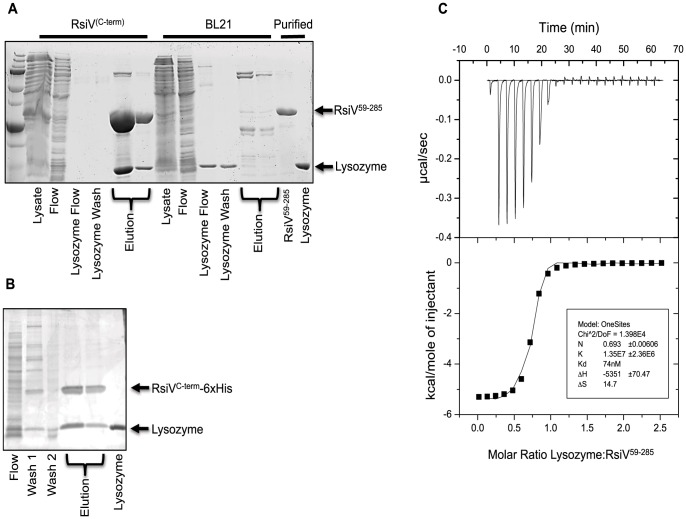
RsiV directly binds lysozyme. **A.** Co-purification experiment showing 6×His-2×Flag-RsiV^59–285^ and HEW lysozyme elute from a Ni affinity column in the same fractions. A mock lysate from BL21 cells containing an empty vector control plasmid did not show this same ability to pull down HEW lysozyme. **B.** *In vivo* lysozyme pull-down and identification. HEW lysozyme binds RsiV *in vivo*. B. *subtilis* strain JLH548 (P_hs_ *-rsiV-6×his* Δ*sigVrsiV::kan*) was grown in the presence of 1 mM IPTG, to induce expression of C-terminally 6×His tagged RsiV. When cultures reached an OD_600_ of 0.8, cells were centrifuged and cell pellets resuspended in protoplast buffer. HEW lysozyme (>98% pure, Sigma) was added and allowed to incubate, at 37°C, for 45 minutes. Supernatant from these cells was then batch purified using Ni resin. Elution fractions show RsiV^59–285^-6×His co-eluting with a protein the same size as lysozyme (HEW lysozyme loaded as size control). Samples from these fractions were transferred to a PVDF membrane, excised, and subjected to Edman Degradation N-terminal sequencing at the Iowa State Proteomics Core to confirm the lower band was HEW lysozyme. **C.** Representative run from isothermal titration calorimetry (ITC) experiments conducted with 6×His-2×Flag-RsiV^59–285^ and HEW lysozyme. A highly specific interaction was observed from these experiments, with a dissociation constant (*K_d_*) of ∼70 nM.

Our data suggest that RsiV is cleaved by the signal peptidase SipS *in vitro* only in the presence of lysozyme. However there is no peptidoglycan present in the *in vitro* reactions which raised the question; why is HEW lysozyme required for site-1 cleavage? We hypothesized that RsiV may directly bind HEW lysozyme. We constructed a 6×His-RsiV^59–285^ fusion and purified it from *E. coli* (Figure S5). We tested the ability of RsiV to bind HEW lysozyme using co-purification. We found that when 6×His-RsiV^59–285^ was bound to the nickel column HEW lysozyme was co-eluted (Figure 3A). However when HEW lysozyme was loaded on a column treated with the extract of BL21(DE3) empty vector-containing cells, we found HEW lysozyme in the flow through and wash fractions but not present in the elution fractions (Figure 3A). To confirm RsiV binds to HEW lysozyme *in vivo* as well as *in vitro* we performed a co-purification experiment using *B. subtilis* producing RsiV^6×His^. We found that when we purified cleaved RsiV^6×His^ after treatment with lysozyme a single band with a similar size to HEW lysozyme co-eluted with RsiV^6×His^ (Figure 3B). We confirmed that this band corresponded to HEW lysozyme by N-terminal sequencing. This suggests that RsiV binds HEW lysozyme both *in vitro* and *in vivo*.

We used Isothermal Titration Calorimetry (ITC) to confirm these observations as well as to determine the affinity of RsiV^59–285^ for HEW lysozyme. We found that RsiV^59–285^ binds to HEW lysozyme in an enthalpically-driven reaction with a *K_d_* of 70 nM (Figure 3C; Table 2). Although it is difficult to determine the precise affinity of HEW lysozyme to peptidoglycan, because peptidoglycan is very heterogeneous, the best data suggest the *K_d_* of PG to lysozyme is ∼50 mM [24]–[26]. This suggests that the HEW lysozyme-RsiV affinity is significantly greater than the affinity of HEW lysozyme for peptidoglycan.

**Table 2:** 

RsiV HEW Lysozyme ITC Summary		
	Average^a^	stdev
*K*	1.44×10^7^	±3.79×10^6^
*K_d_*	70 nM	±10 nM
*N*	0.67	±0.01
*ΔH*	−5.2 kcal/mol	±0.1
*-TΔS*	−4.6 kcal/mol	±1.2

a Average values obtained from three independent experiments.

### Muramidase Activity Is Not Sufficient for σ^V^ Activation

**Figure 4:**
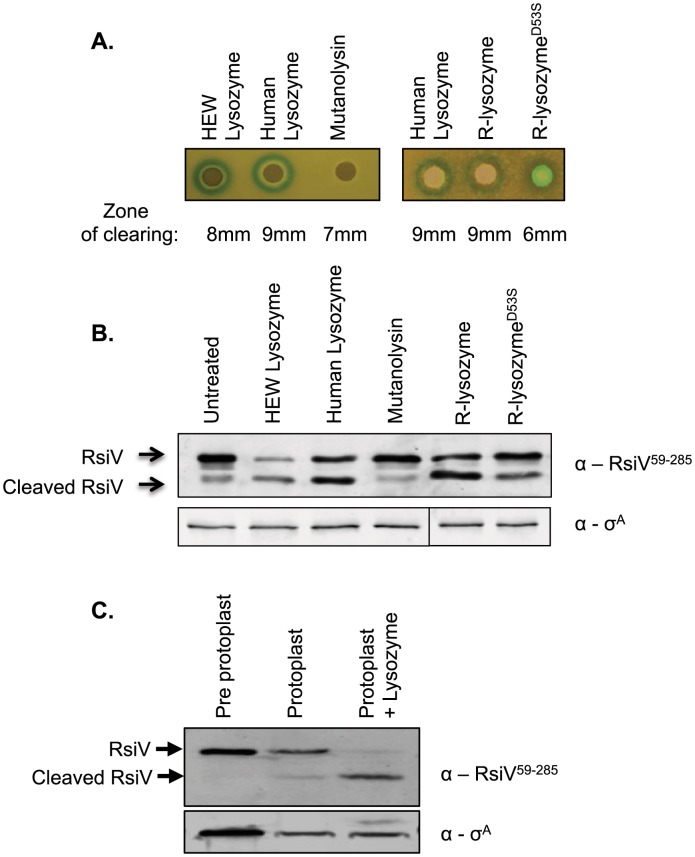
Muramidase activity is not sufficient to activate σ^V^. **A.** Bacteria containing the P*_sigV_*-*lacZ* transcriptional fusion (CDE1546) were grown to an OD_600_ of 1, and then diluted 1∶100 in 3 ml of top agar containing 200 µg/ml X-gal and spread on an LB X-gal plate. Filter disks containing 10 µl of 20 mg/ml HEW lysozyme, HUM lysozyme, mutanolysin, or 10 µl of 4 mg/ml HUM lysozyme, R-lysozyme, R-lysozyme^D53S^ were placed on the top agar and incubated 16 hours at 37°C. **B.** *B. subtilis* containing P*_hs_-rsiV^+^* (JLH402) were subcultured from an overnight culture 1∶100 into LB+1 mM IPTG and grown to an OD_600_ of 0.8. Then muramidases were added to the cultures at the following final concentrations HEW lysozyme (2 µg/ml), HUM lysozyme (2 µg/ml), mutanolysin (2 µg/ml), R-lysozyme (4 µg/ml), or R-lysozyme^D53S^ (4 µg/ml) and incubated for 10 minutes at 37°C. Cells were collected by centrifugation and resuspended in 100 µl Laemmli sample buffer. Immunoblot was probed with anti-RsiV antibodies. Sigma A was used as an internal loading control probed using anti-σ^A^. **C.** *B. subtilis* containing P*_hs_*-*rsiV^+^* (JLH402) was subcultured from an overnight culture 1∶100 into LB+1 mM IPTG and grown to an OD_600_ of 0.8–1. Cells were collected by centrifugation and resuspended in 100 µl 2× Laemmli sample buffer (lane 1) or washed with 1× PBS and resuspended in protoplast buffer (lanes 2 and 3). Mutanolysin was added to a final concentration of 2 µg/ml, and incubated shaking at 37°C for 40 minutes to form protoplasts. Samples were either left untreated or treated with 2 µg/ml lysozyme for 10 minutes. Immunoblot analysis with anti-RsiV antibodies.

Previous work found that lysozyme was able to induce σ^V^ activity. In contrast, a variety of antimicrobial compounds that inhibited peptidoglycan synthesis and damaged the cell envelope were unable to induce σ^V^ activity [9]. Since RsiV binds HEW lysozyme, we hypothesized that the protein and not the activity of HEW lysozyme was required to activate σ^V^. Mutanolysin cleaves the same β-glycosidic bond as HEW lysozyme, but has an entirely different amino acid sequence and structure [27]. To determine if muramidase activity was sufficient for σ^V^ activation we compared the ability of HEW lysozyme, human lysozyme, and mutanolysin to induce expression of the P*_sigV_-lacZ* reporter fusion. We found that HEW lysozyme and human lysozyme, both C-type lysozymes with 58% amino acid identity and 76% similarity, produced zones of clearing (8 mm and 9 mm respectively), and induced expression of P*_sigV_-lacZ* as indicated by the blue ring around the disk (Figure 4A). We found that although mutanolysin produced a zone of clearing (7 mm) indicative of killing, it was unable to induce P*_sigV_-lacZ* expression. The lack of induction by mutanolysin suggests that muramidase activity is not sufficient for activation of σ^V^.

Since σ^V^ is activated upon degradation of the transmembrane bound anti-σ RsiV we tested the effect of HEW lysozyme, human lysozyme, and mutanolysin, for their ability to induce RsiV degradation. We expressed *rsiV* from an IPTG inducible promoter and then treated cells with a sub-lethal concentration of lysozyme for 10 minutes. Using anti-RsiV^59–285^ antibodies a 32 kDa protein was observed by immunoblot in the absence of lysozyme, indicative of the full length RsiV (Figure 4B). In the presence of HEW lysozyme, the full-length RsiV is rapidly degraded and a smaller product, the released extracellular domain, is visible (Figure 4B). Similarly, treatment with human lysozyme also induces RsiV degradation (Figure 4B). However, treatment with mutanolysin was unable to induce degradation of RsiV (Figure 4B).

To further confirm the observation that muramidase activity was insufficient to induce RsiV degradation, we used mutanolysin to generate protoplasts of *B. subtilis* cells expressing *rsiV*. These cells were then left untreated or treated with HEW lysozyme and the status of RsiV monitored by immunoblot. We found that the protoplasts still retain full-length RsiV however upon subsequent treatment with lysozyme we observe loss of full-length RsiV (Figure 4C). This provides further evidence that muramidase activity alone is not sufficient to induce degradation of RsiV.

### Muramidase Deficient Lysozyme Activates σ^V^

Our data indicate that muramidase activity provided by mutanolysin is not sufficient to induce σ^V^ activation or RsiV degradation, thus we asked if muramidase activity was required for lysozyme-driven σ^V^ activation or RsiV degradation. It has been shown that changing human lysozyme aspartate 53 to serine abolishes muramidase activity to less than 1% [28]. Recombinant human lysozyme (R-lysozyme), and the catalytically inactive form of human lysozyme (R-lysozyme^D53S^) were expressed and purified from the supernatant of *Pichia pastoris* [29]. Muramidase activity of R-lysozyme and R-lysozyme^D53S^ was assayed against *Micrococcus lysodekticus* peptidoglycan which confirmed that the R-lysozyme was active and R-lysozyme^D53S^ was muramidase deficient (Figure S6).

We found that when purified R-lysozyme and R-lysozyme^D53S^ were placed on a lawn of *B. subtilis* containing the P*_sigV_-lacZ* reporter fusion both produced a zone of induction (Figure 4A). However only the wild type R-lysozyme produced a zone of clearing (9 mm) while the R-lysozyme^D53S^ did not produce a zone of clearing (6 mm – size of the disk) (Figure 4A). To further confirm that muramidase activity was not required for σ^V^ activation we tested the ability of the recombinant active or inactive lysozyme to induce RsiV degradation. Consistent with the zone of clearing results, treatment with human lysozyme, R-lysozyme, and R-lysozyme^D52S^ also induce RsiV degradation (Figure 4B). However, treatment with mutanolysin was unable to induce degradation of RsiV (Figure 4B). Thus, the lack of P*_sigV_-lacZ* induction by mutanolysin and the ability of catalytically inactive R-lysozyme^D53S^ to induce P*_sigV_-lacZ* suggests that muramidase activity is not required nor is it sufficient for activation of σ^V^.

### RsiV Binds C-Type Lysozymes HEW Lysozyme and Human Lysozyme, but Not Mutanolysin

**Figure 5:**
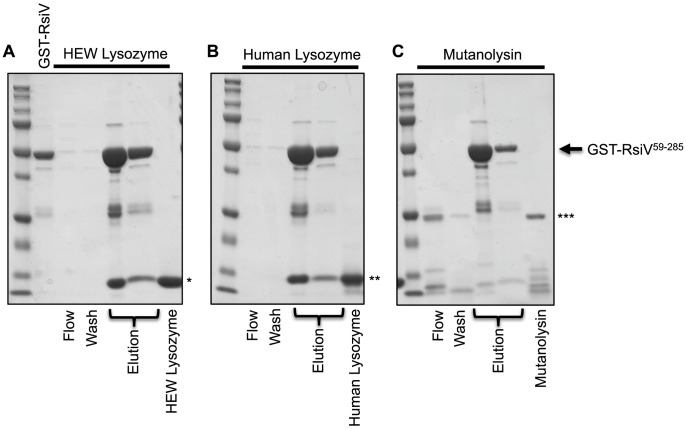
RsiV binds C-type lysozyme but not mutanolysin. 2 mg of purified GST-RsiV^59–285^, in PBS-EW, was loaded on a Glutathione HiCap Matrix column. 2 mg of HEW lysozyme (**A**), Human lysozyme (**B**), or mutanolysin (**C**), reconstituted in PBS-EW, was applied to the column. PBS-EW buffer and TNGT buffer were used as wash and elution buffers, respectively. Samples were taken and analyzed by SDS-PAGE as described for the 6×His-tagged proteins above. (*HEW lysozyme, **Human lysozyme, ***Mutanolysin, arrow indicates where GST-RsiV^59–285^ migrates on the gel).

Our data suggests that σ^V^ is activated by the C-type lysozymes HEW lysozyme and human lysozyme, but not the unrelated muramidase mutanolysin. Since RsiV can bind HEW lysozyme we sought to determine if σ^V^ activation was correlated with the ability of RsiV to bind different proteins. To test this we purified a GST-RsiV^59–285^ fusion protein. We then loaded 2 mg of GST-RsiV^59–285^ onto a glutathione column and then 2 mg of either HEW lysozyme, human lysozyme, or mutanolysin were passed over the column. The proteins were eluted and separated by SDS/PAGE (Figure 5). We found that both HEW lysozyme and human lysozyme were retained on the column when RsiV was present. This suggests that both HEW lysozyme and human lysozyme can bind RsiV^59–285^. In contrast, we found when mutanolysin was loaded onto the GST-RsiV-RsiV^59–285^ containing column mutanolysin was collected almost entirely in either the flow through and wash fractions (Figure 5). This suggests that RsiV specifically binds C-type lysozymes but not mutanolysin.

### *C. difficile* and *E. faecalis* RsiV Directly Bind HEW Lysozyme

**Figure 6:**
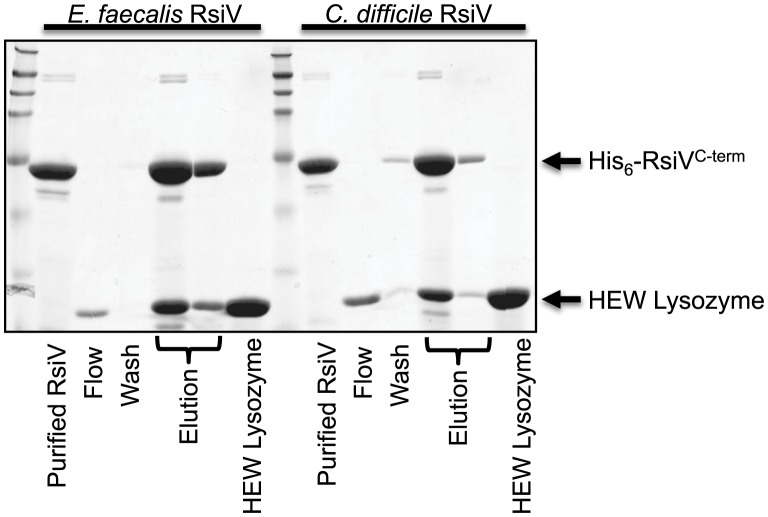
Extracellular domain of RsiV from *C. difficile* and *E. faecalis* RsiV bind lysozyme. Samples from a lysozyme pull-down assay were separated on a 15% SDS-PAGE gel and stained with Coomassie brilliant blue. The pull down assay was performed as described in Materials and Methods. Recombinant 6×His-RsiV^C-term^ from both *E. faecalis* (6×His-RsiV*^72–294^*) and *C. difficile* (6×His-RsiV^69–298^) were used in this assay. Elution fractions for both of these proteins show both RsiV^C-term^ and HEW lysozyme co-eluting from the Ni-NTA column. Purified RsiV^C-term^ from each organism and HEW lysozyme alone are shown for comparison. Arrows indicate where RsiV^C-term^ and HEW Lysozyme migrate.

Both *C. difficile* and *E. faecalis* encode homologs of σ^V^ and RsiV and in each organism σ^V^ is activated by lysozyme and required for lysozyme resistance [11], [13], [30]. To determine if the ability of the anti-σ factors to interact with lysozyme was a conserved feature we purified the histidine-tagged extracellular domains of both *C. difficile* RsiV^69–289^ (RsiV^CD^) and *E. faecalis* RsiV^72–294^ (RsiV^EF^) and conducted binding assays. Briefly, purified *C. difficile* RsiV^CD^ or *E. faecalis* RsiV^EF^ protein was loaded onto a nickel column and 2 mg of HEW lysozyme passed over the column bound protein. We found that columns containing either *C. difficile* or *E. faecalis* RsiV resulted in retention of HEW lysozyme on the column. Upon elution from the column, we found that HEW lysozyme co-eluted with both RsiV^CD^ and RsiV^EF^ (Figure 6). This suggests that the ability of the anti-σ factor, RsiV, to bind lysozyme is a conserved feature present in RsiV homologs from other species.

## Discussion

### RsiV as a Receptor for C-Type Lysozyme

The primary observations of this work are that the anti-σ factor and RIP substrate RsiV acts as a sensor for the presence of lysozyme and that SipS functions as a site-1 protease. This hypothesis is supported by the following observations 1) Co-purification experiments in which RsiV binds both human and HEW lysozyme, 2) ITC showing direct binding of RsiV and HEW lysozyme, 3) RsiV cleavage *in vitro* at site-1 by SipS only in the presence of lysozyme, 4) Insufficiency of muramidase activity for σ^V^ activation and RsiV degradation and 5) Induction of σ^V^ activation and RsiV degradation by catalytically inactive human lysozyme. Taken together this data supports a model in which RsiV is a receptor for lysozyme.

We identify the signal peptidase SipS as a site-1 protease for the anti-σ factor RsiV. The evidence to support signal peptidase as the site-1 protease for RsiV is as follows. The site-1 cleavage site of RsiV was identified and found to resemble a signal peptide cleavage site. Mutating this cleavage site blocks RsiV degradation and σ^V^ activation. Using a cell free transcription/translation system, we demonstrate *in vitro* that SipS was sufficient for site-1 cleavage of RsiV only in the presence of lysozyme. Together these data indicate that signal peptidase is the site-1 protease for RsiV. The signal peptidases of *B. subtilis* are redundant, but cells lacking both SipS and SipT are not viable [20]. RsiV was found to be cleaved at site-1 in the absence of SipS or SipT. Thus we hypothesize that one or more of the *B. subtilis* signal peptidases can cleave RsiV at site-1 *in vivo*.

Previous work from our lab found that σ^V^ was activated only by lysozyme and not by other cell envelope stresses [9]. Here we showed that muramidase activity was not required nor was it sufficient to activate σ^V^ or induce RsiV degradation. In fact, we did not detect significant cleavage of RsiV in *B. subtilis* protoplasts generated by mutanolysin. However, upon addition of HEW lysozyme to these protoplasts RsiV was rapidly cleaved at site-1. In addition, the catalytically inactive form of lysozyme (R-lysozyme^D53S^) was still able to activate σ^V^ and degrade RsiV. Finally, cleavage of RsiV by SipS *in vitro* was dependent upon HEW lysozyme suggesting RsiV binding to lysozyme is required for RsiV cleavage independent of muramidase activity.

**Figure 7:**
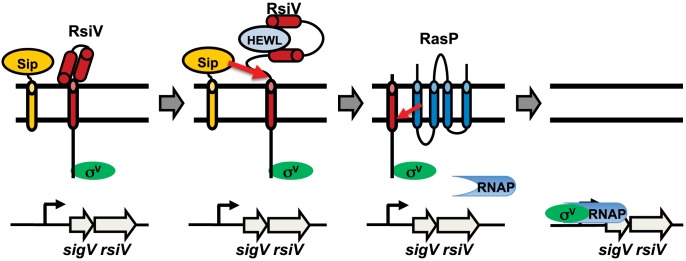
Model of σ^V^ activation. Shown in green is σ^V^, the anti-σ factor RsiV is red, the signal peptidase is orange, the site-2 protease RasP is dark blue and lysozyme is in light blue. In the absence of lysozyme RsiV inhibits σ^V^ activity and is resistant to cleavage by signal peptidase. Upon binding to lysozyme RsiV becomes sensitive to signal peptidase and is cleaved at site-1 by signal peptidase. The site-1 cleaved RsiV then becomes a substrate for the site-2 protease RasP. This frees σ^V^ to interact with RNA polymerase and transcribe target genes.

Based upon these observations we propose a model of σ^V^ activation in which RsiV is a receptor for the C-type lysozymes, HEW lysozyme and human lysozyme (Figure 7). In the absence of C-type lysozyme RsiV is in a conformation which is resistant to signal peptidase and RsiV inhibits σ^V^ activity by sequestering it to the membrane (Figure 7). In the presence of C-type lysozyme, the C-terminal domain of RsiV binds to lysozyme (Figure 7). When RsiV is bound to lysozyme, RsiV undergoes a conformational change, which allows signal peptidase to cleave RsiV at site-1 (Figure 7). This allows the site-2 protease to cleave the truncated form of RsiV leading to release of the RsiV-σ^V^ complex (Figure 7) [14]. The cytoplasmic portion of RsiV is then presumably degraded by cytosolic proteases, resulting in free σ^V^ which can complex with RNA polymerase and transcribe genes required for lysozyme resistance (Figure 7) [9], [10].

There are several examples of anti-σ factors directly sensing inducing signals, however in these cases the anti-σ factors are not degraded in RIP dependent manner. For example the anti-σ factor of *Streptomyces coelicolor* σ^R^, RsrA, is responsible for sensing redox stress [31]–[33] and the anti-σ factor of *Rhodobacter sphaeroides* σ^E^, ChrR, is responsible for sensing singlet oxygen [34], [35]. In *E. coli* regulation of the genes encoding the FecABCDE iron transport system are controlled by the iron responsive FecRI system. Evidence suggests that the anti-σ factor FecR can act indirectly as a sensor for the presence of iron-citrate [36]. However, to our knowledge this is the first example of a RIP controlled anti-σ factor acting as a receptor for the inducing signal. In the most well studied cases, the ECF σ factors that are controlled by RIP, it is the site-1 proteases that are the sensors for cell envelope damage. In particular it is clear that activation of σ^E^ in *E. coli* requires binding of unfolded outer membrane β-barrel proteins to the site-1 protease DegS, which allows DegS to cleave the σ^E^ anti-σ factor RseA [4], [6], [7]. In addition to DegS, the RseA binding protein, RseB, also contributes to sensing cell envelope stress [37]–[39]. In the case of σ^W^ activation in *B. subtilis*, isolation of mutations in the site-1 protease PrsW which result in constitutive degradation of RsiW again suggest the protease itself is the sensor for the inducing signal [8]. We found that SipS was able to cleave RsiV at site-1 only in the presence of HEW lysozyme. The enzymatic activity of signal peptidase is not known to be regulated by environmental signals. Since lysozyme binds RsiV we hypothesize that the mechanism for controlling site-1 cleavage of RsiV doesn't reside with the site-1 protease, but within the anti-σ factor itself.

### A Role for Signal Peptidase in Signal Transduction

There are several examples of signal peptidases being required for production of quorum sensing signals. For example, in *S. aureus* signal peptidase is required for production of the quorum sensing peptide or auto-inducing peptide AIP [40]. In *B. subtilis* signal peptidases are required for production of the quorum sensing Phr peptides [41]. Once the Phr peptides released from the cell accumulate to sufficient quantities they are thought to directly or indirectly inhibit the Rap phosphatases which control initiation of sporulation and competence processes [42]–[44]. In each of these cases however the role of signal peptidase is not in sensing of a signal but in the production of a signal.

There is some recent evidence that signal peptidases may be involved in signal input by cleaving a sensor for detecting β-lactam antibiotics. In *Staphylococcus epidermidis* it has been observed that the β-lactam sensor domain of BlaR1 (a protease which degrades the transcription regulator BlaI) was released in what appears to be a signal peptidase dependent process [45]. Similarly, in *Staphylococcus aureus* there is recent evidence to suggest that this β-lactam sensor domain is also removed at what appears to be a putative signal peptide cleavage site [46]. However it is not yet clear what impact this processing has on the signal transduction activity of BlaR1.

### σ^V^ Activation and RsiV Degradation in Other Organisms

Homologs of the σ^V^ system in *E. faecalis* and *C. difficile* are also induced by lysozyme [11], [13], [30]. In both of these organisms σ^V^ activity is inhibited by RsiV [11], [30]. We found that the RsiV homologs from both *C. difficile* and *E. faecalis* also bind HEW lysozyme, suggesting RsiV from these organisms may also sense lysozyme directly and activate using a similar mechanism. Although the site-1 protease that initiates RsiV degradation in *E. faecalis* or *C. difficile* is not known, an alignment of 185 RsiV homologs using MEME [17] reveals a highly conserved AXA motif near the predicted transmembrane domain of these RsiV homologs. Furthermore analysis of *C. difficile*, *E. faecalis* and *B. subtilis* RsiV homologs using SignalP [16] reveals potential signal peptidase cleavage sites in each RsiV homolog (Figure S2A). Future work will be required to see if site-1 cleavage of RsiV in other organisms is also carried out by signal peptidase.

Activation of σ^V^ in *B. subtilis* and *E. faecalis* requires cleavage of RsiV by a site-2 protease [14], [47]. In addition to cleavage of RsiV and other anti-σ factors, RasP is also able to clear signal peptidase processed signal peptides from the membrane [48]. Thus, it appears that the σ^V^-RsiV signal transduction system has “plugged into” the signal peptide processing system. Interestingly, the σ^V^-RsiV signal transduction system is present primarily in many, but not all firmicutes, suggesting the σ^V^–RsiV system may be transmitted by horizontal gene transfer. Thus the ability of the anti-σ factor to act as a sensor and capitalize on an essential system present in all bacteria, may provide a mechanism to control activation of horizontally acquired ECF σ factors.

One of the surprising findings is the specific activation of σ^V^ by C-type lysozyme but not mutanolysin. In *B. subtilis* σ^V^ holo RNA polymerase transcribes *oatA* which encodes an O-acetyl transferase and in *C. difficile* and *E. faecalis* σ^V^ holo RNA polymerase transcribes a peptidoglycan deacetylase gene [9], [13], [49], [50]. Interestingly both mechanisms increase resistance to C-type lysozymes (HEW and human lysozyme) [50]–[54] but not mutanolysin [55]–[57]. Thus rather than relying on peptidoglycan damage or cell envelope stress the RsiV- σ^V^ system appears to have evolved to respond to C-type lysozyme through a receptor-ligand interaction. This interaction induces genes which provide resistance to C-type lysozyme but not to other muramidases.

An interesting question raised by the ability of *B. subtilis* to respond specifically to C-type lysozymes is when does *B. subtilis* encounter these factors. *B. subtilis* is often viewed simply as a soil organism however recent evidence suggests that it can colonize the intestinal tracts of a number of different organisms including *Drosophila melanogaster*, chickens, mice and humans [58]–[61] all of which encode C-type lysozymes [62]. Thus while *B. subtilis* is primarily a soil organism it may also have a more complex life associated with intestinal tract of a diverse number of organisms. It is tempting to hypothesize that lysozyme resistance could be an important trait required for colonization of the intestinal tract of higher organisms.

## Materials and Methods

### Strain Construction

Strains are isogenic derivatives of PY79, a prototrophic derivative of *B. subtilis* strain 168, and are listed in Table 3 [63]. *B. subtilis* competent cells were prepared by the one-step method previously described [64]. All plasmid constructs are listed in Table 4 were confirmed by DNA sequencing (University of Iowa). All oligonucleotides are listed in Table S1.

**Table 3:** Strain list.

Strain	Genotype^a^	Reference^b^
PY79	Prototrophic derivative of *B. subtilis* 168	[63]
CDE1546	PY79 *pyrD*::P*_sigV_-lacZ* (*cat*)	[9]
CDE1563	PY79 Δ*sigVrsiV::kan*	[14]
CDE2379	PY79 *pyrD*::P*_sigV_-lacZ* (*cat*) *rsiV*::*rsiV^A66W^*	
JLH402	PY79 *amyE*::P*_hs_-rsiV^+^(spec)* Δ*sigVrsiV::kan*	[14]
JLH623	PY79 *amyE*::P*_hs_-rsiV^A66W^(spec)* Δ*sigVrsiV::kan*	
JLH548	PY79 *amyE*::P*_hs_-rsiV-6×his* Δ*sigVrsiV::kan*	
JLH708	PY79 *amyE*::P*_hs_-3×flag-CBP-rsiV* Δ*sigVrsiV::kan*	
JLH933	PY79 *amyE*::P*_hs_-rsiV^+^(spec)* Δ*sigVrsiV::kan* Δ*sipS*::*cat*	
JLH953	PY79 *amyE*::P*_hs_-rsiV^+^(spec)* Δ*sigVrsiV::kan* Δ*sipT::tet*	
GS115	*P. pastoris his4*	Invitrogen
JLH1056	*P. pastoris* P_AOX1_-*hlyz*	
JLH1057	*P. pastoris* P_AOX1_-*hlyz* ^D53S^	
BL21(DE3)	*E. coli fhuA2 [lon] ompT gal (λ DE3) [dcm]* Δ*hsdS λ DE3 (sBamHIo* Δ*EcoRI-B int::(lacI::PlacUV5::T7 gene1) i21* Δ*nin5)*	

a All strains are isogenic derivatives of PY79.

b This study, unless otherwise noted.

**Table 4:** Plasmid list.

Strain/Plasmid	Genotype	Reference Source^a^
pDR111	*amyE* P*_hs_ specR ampR* P_hs_	[80]
pCE292	pDR111 *amyE* P*_hs_ specR ampR* P*_hs_*-RfA (*ccdB^+^ camR*)	[11]
pCE458	pEntrD-topo *rsiV* ^59–285^ *kanR*	[14]
pMZS3F	SPA tag *ampR*	[68]
pCE417	*amyE* P*_hs_ specR ampR* P*_hs_*-*3×flag*-*cbp*	
pCE418	*amyE* P*_hs_ specR ampR* P*_hs_*-*3×flag*-*cbp-RfC.1*	
pCE352	pEntrD-topo N-term fusion ‘*rsiV^+^*	
pCE422	*amyE* P*_hs_ specR ampR* P*_hs_*-*3×flag*-*cbp-rsiV^+^*	
pMAD	*bla ermC* ori pBR322 ori pE194^ts^	[65]
pCE492	pMAD *sigV* ^+^ *rsiV^A66W^*	
pJH219	pEntrD-topo *rsiV^A66W^*	
pJH224	pCE292 *amyE* P*_hs_ specR ampR* P_hs_-*rsiV^A66W^*	
pKBW101	pEntrD-topo *‘2×flag-rsiV^59–285^ kanR*	
pEU-Flexi-His	P*_sp6_ ampR catR sacB*	[81]
pCE455	pEU-Flexi-His P*_sp6_*-*3×flag*-*cbp*-*rsiV*-*6×his ampR*	
pCE448	pEU-Flexi-His P*_sp6_*-*sipS ampR*	
pCE490	pEU-Flexi-His P*_sp6_*-*3×flag*-*cbp*-*rsiV^A66W^*-*6×his ampR*	
pDEST15	*P_T7_GST cat ccdB ampR* ori pBR322	Invitrogen
pDEST17	*P_T7_6×his cat ccdB ampR* ori pBR322	Invitrogen
pKBW204	pDEST15 P_T7_-GST-*rsiV^59–285^ ampR*	
pKWB201	pDEST17P_T7_ *-6×his-2×flag*-*rsiV* ^59–285^	
pCE363	pEntrD-topo *rsiV-6×his kanR*	
pJH215	*amyE* P*_hs_ specR ampR* P*_hs_*-*rsiV-6×his*	
pDG1515	*ampR tetR*	[82]
pDG1661	*amyE lacZ ampR catR*	[83]
pJH228	pEntrD-topo *rsiV^EF72–294^ kanR*	
pJH227	pDEST17 P_T7_-*6×his-rsiV^EF72–294^ ampR*	
pCE302	pEntrD-topo *rsiV^CD69–298^ kanR*	
pKBW216	pDEST17 P_T7_-*6×his-rsiV^CD69–298^ ampR*	
pICzα	P_AXO1_ –α factor signal sequence *zeoR*	Invitrogen
pJH326	pICzα h*lyz zeoR*	
pJH327	pICzα h*lyz* ^D53S^ *zeoR*	

a This study, unless otherwise noted.

The *rsiV^A66W^* mutation was introduced onto the chromosome of PY79 by homologous recombination using the temperature sensitive plasmid pMAD [65]. To clone *rsiV^A66W^* we PCR amplified *rsiV^A66W^* plus ∼1 kb upstream with CDEP1892 and CDEP1562 and *rsiV^A66W^*+∼1 kb downstream using CDEP1561-CDEP1893. The resulting PCR products were cloned into pMAD digested with SmaI using Isothermal assembly [66]. The resulting plasmid, pCE492, was transformed into PY79 and the *rsiV^A66W^* mutation was introduced onto the chromosome by shifting temperatures as previously described [65]. The presence of the *rsiV^A66W^* mutation was confirmed by sequencing *rsiV*.

Site-directed mutagenesis of *rsiV* was performed using the QuickChange site-directed mutagenesis kit (Agilent Technologies). The *rsiV^A66W^* mutation was constructed using primer pairs CDEP1561 and CDEP1562 to generate plasmid pJH219. For IPTG-inducible expression, the *rsiV* ^A66W^ mutant was moved into pCE292 [11] using LR Clonase II (Invitrogen). The resulting plasmid, pJH224, places the IPTG-inducible hyper-spank (P*_hs_*) promoter upstream of *rsiV* ^A66W^ and was transformed into *B. subtilis* CDE1563 to result in JLH623.

C-terminal 6×His tagged *rsiV* with an optimized ribosome binding site was PCR amplified from *B. subtilis* using oligos CDEP1544 and CDEP1430 cloned into pEntrD-TOPO (Invitrogen) resulting in pCE363. For IPTG-inducible expression, the *rsiV-6×his* was moved into pCE292 using LR Clonase II resulting in pJH215. The plasmid pJH215 was transformed into CDE1563 resulting in JLH548.

A vector for a 3×Flag-CBP was constructed by PCR amplification of the gene encoding the calmodulin binding peptide (CBP) from pMZS3F with CDEP1611 and CDEP1610 [67], [68]. The resulting PCR was amplified with CDEP1612 and CDEP1610. The PCR was digested with HindIII and SphI and ligated into pDR111 digested with the same enzymes resulting in pCE417. The pCE417 was converted to a Gateway destination vector by cloning the RfC.1 cassette into pCE417 digested with Eco53kI resulting in pCE418. To construct a plasmid producing 3×Flag-cbp-RsiV^+^, *rsiV* ^+^ was moved from pCE352 onto pCE418 using LR Clonase II resulting in plasmid pCE422.

Vectors for cell free production of RsiV and SipS production were constructed by cloning the genes on a plasmid downstream of an SP6 promoter. The *sipS* gene was PCR amplified from *B. subtilis* using CDEP1678 and CDEP1679. The PCR product was digested with AsiSI and SmaI and cloned into pEU-Flexi-His digested with AsiSI and PmiI using T4 ligase resulting in pCE448. The *3×flag-cbp-rsiV^6×his^* gene was PCR amplified from pCE422 using CDEP1677 and CDEP1714. The PCR product was digested with AsiSI and SmaI and cloned into pEU-Flexi-His digested with AsiSI and PmiI using T4 ligase resulting in pCE455. The *3×flag-cbp-rsiV^A66W 6×his^* for cell free production of RsiV^A66W^ was created by QuikChange mutagenesis resulting in pCE490.

The human lysozyme open reading frame was synthesized by GenScript and the codons were optimized for expression in yeast. Using primers CDEP1888 and CDEP1889 human lysozyme was PCR amplified and cloned into *P. pastoris* expression vector pICZα (Invitrogen) by isothermal assembly [66] resulting in pJH326. To produce inactive lysozyme a mutation was constructed to change the codon for aspartate 53 to serine (lysozyme^D53S^) using primers CDEP1847 and CDEP1848 and PCR QuickChange site-directed mutagenesis (Agilent Technologies) resulting in pJH327. Expression vectors pJH326 and pJH327 were transformed into *P. pastoris* GS115 via the lithium chloride method (Invitrogen [69]) resulting in JLH1056 and JLH1057 respectively. Appropriate integration of the plasmids was confirmed by zeocin resistance and PCR using the primers CDEP1888 and CDEP1889.

For purification of *E. faecalis* RsiV, the portion of *rsiV* encoding the C-terminal extracellular domain (*rsiV^EF^*) was PCR amplified using CDEP1434 and CDEP1435. The PCR product was cloned into pEntrD-TOPO resulting in pJH228. To tag *E. faecalis* RsiV with 6×His, *rsiV^EF^*, was moved into pDEST17 using LR Clonase II resulting in pJH227.

For purification of *C. difficile* RsiV the portion of the *rsiV* encoding the C-terminal extracellular domain (*rsiV^CD^*) was PCR amplified using CDEP928 and CDEP189. The PCR product was cloned into pEntrD-TOPO resulting in pCE302. To tag *C. difficile* RsiV with 6×His, *rsiV^CD^*, was moved into pDEST17 using LR Clonase II, resulting in pKBW216.

To tag *B. subtilis* RsiV with GST, *rsiV* ^59–285^, was moved from pCE458 [14] into pDEST15 using LR Clonase II resulting in pKBW204.

Construction of 2×flag-*rsiV^59–285^* was performed by PCR amplification from *B. subtilis* genomic DNA using CDEP1140 and CDEP952. The resulting product was used as template in another round of PCR using CDEP950 and CDEP952 and cloned into pEntrD-TOPO, resulting in pKBW101. This was moved into the IPTG inducible 6×His expression vector pDEST17 using LR Clonase II, resulting in pKBW201.

*ΔsipS::cat* and *ΔsipT::tet* were constructed using long flanking homology PCR. Briefly *sipS* flanking regions were constructed by PCR amplifying with CDEP1697-CDEP1709 and CDEP1710-CDEP1700. The *sipT* flanking regions were constructed by PCR amplifying with CDEP1701-CDEP1711 and CDEP1712-CDEP1704. The resulting PCR products were used as primers to amplify either a chloramphenicol antibiotic cassette generated by PCR from pDG1661 using (CDEP1954-CDEP1955) or tetracycline antibiotic cassette from either pDG1515 respectively. The PCR products were transformed into *B. subtilis* JLH402 (*amyE*::P*_hs_-rsiV^+^(spec) ΔsigVrsiV::kan*) and confirmed by PCR resulting in JLH933 and JLH953 respectively.

### Medium Supplements

Antibiotics were used at the following concentrations: chloramphenicol, 5 µg/ml; erythromycin plus lincomycin, 1 µg/ml and 25 µg/ml; kanamycin, 5 µg/ml; spectinomycin, 100 µg/ml; tetracycline, 10 µg/ml; ampicillin 100 µg/ml. The β-galactosidase chromogenic indicator 5-bromo-4-chloro-3-indolyl β-D-galactopyranoside (X-Gal) was used at a concentration 100 µg/ml. Isopropyl β-D-1-thiogalactopyranoside (IPTG) was used at a final concentration of 1 mM unless otherwise noted.

### Purification of Site-1 Cleavage Product from *B. subtilis*

Cells producing RsiV-6×His were grown to an OD of 1 and then subcultured 1∶100 into 1L LB+IPTG (1 mM) and grown to an OD of 0.8. Cells were pelleted by centrifugation at 5000× *g* and frozen at −80°C. Cell pellets were thawed on ice and resuspended in 20 mL protoplast buffer (0.4 M sucrose, 10 mM potassium phosphate, 15 mM MgCl_2_) [70]. HEW lysozyme was added to a final concentration of 8 µg/ml and the cells were incubated at 37°C for 45 minutes. Protoplast formation was confirmed by phase contrast microscopy. Cells were pelleted by centrifugation at 5000× *g* and RsiV-6×His was purified from the supernatant using Ni resin (Thermo-Fisher). The resulting protein was separated by SDS PAGE and transferred to PVDF membrane (BioRad). The band containing the protein of interest was confirmed by immunoblot with anti-RsiV^59–285^ antibodies (Figure S1). The band of interest was cut out from the membrane and submitted for Edman degradation analysis (Iowa State University).

### β-Galactosidase Activity Assays

Cultures were grown overnight in LB broth at 30°C and 20 µl were spotted onto LB agar + 10 µg/ml lysozyme. Plates were incubated at 37°C for 6 hours. Cells were harvested and resuspended in 500 µl of Z buffer (60 mM Na_2_HPO_4_, 40 mM NaH_2_PO_4_, 10 mM KCl, 1 mM MgSO_4_, 50 mM β-mercaptoethanol pH 7.0). Cells were transferred to a 96 well plate and optical density (OD_600_) determined. Cells were permeabilized by mixing with chloroform and 2% sarkosyl [9], [71]. Permeabilized cells (100 µl) were mixed with 10 mg/ml ortho-Nitrophenyl-β-galactoside (ONPG, RPI, 50 µl) and OD_405_ was measured over time. β-galactosidase activity units (µmol of ONP formed min^−1^)×10^3^/(OD_600_×ml of cell suspension) were calculated as previously described [72]. Experiments were performed in triplicate. Mean and standard deviation are shown.

### Lysozyme Sensitivity Assays

To determine lysozyme MIC values, *B. subtilis* strains were grown 16 h at 30°C and then subcultured 1∶100 in LB. Cells (100 µl) were inoculated into 100 µl of two fold serial dilutions of HEW lysozyme ranging from 200 µg/ml to 0.15 µg/ml in a round-bottom 96 well plate. The absorbance at OD_600_ was taken every 30 minutes using a Tecan F50 (Tecan) over a period of 20 hrs. Growth was defined as an OD_600_ greater than 0.1 at 14 hrs.

To determine lysozyme zones of clearing, *B. subtilis* strains were grown 16 h at 30°C and then diluted 1∶100 in 1.5 mL LB top agar (0.75%) containing X-Gal (100 µg/ml). Top agar was spread on solid LB+X-Gal and allowed to solidify. Whatman filter disks containing 10 µl of 10 mg/ml HEW lysozyme were placed on the top agar and incubated 16 h at 37°C.

### Transcription and Cell-Free Translation Reactions

Plasmids were purified using QIAprep spin columns according to the manufacturer's instructions and resuspended in 30 µl of Milli-Q water. The concentration of the RNase-free plasmid DNA was determined by absorbance at 260 nm.

Small-scale transcription reactions were performed as previously described [21]. Briefly, the reaction mixtures were composed of 80 mM HEPES-KOH pH 7.8, 2 mM magnesium acetate, 2 mM spermidine, 10 mM DTT, 4 mM of each nucleotide triphosphate (ATP, CTP, UTP and GTP), 1.6 U/µl SP6 RNA polymerase (Promega), 1 U/µl RNasin (Promega) and 0.2 mg/mL of RNase-free plasmid DNA in a 10 µl total reaction volume. Each sample was incubated at 37°C for 4 h. The mRNA from each transcription reaction (5 µl) was mixed with 20 µl of the translation mix composed of 25% v/v wheat germ extract (WEPRO 2240 Cell free Sciences, Yokohama, Japan), 13 mM HEPES-KOH pH 7.8, 55 mM KOAc, 1.7 mM Mg(OAc)_2_, 0.22 mM spermidine hydrochloride, 2.2 mM DTT, 0.7 mM ATP, 0.14 mM GTP, 9 mM creatine phosphate, 0.003% NaN_3_, 1 mg/mL creatine kinase and 2 mM amino acids. The reaction mixtures were added to 12-kDa MWCO dialysis cups. The reservoir buffer contained all the reagents listed above except RNasin and wheat germ extract as previously described [21]. Each reaction was incubated for 16 h at room temperature.

### *In Vitro* Protease Activity of SipS

The pellet fractions from the above-described translation reactions for SipS, RsiV (full-length), and RsiV^A66W^ were resuspended in 5 mM MES pH 7.0, 50 mM NaCl and mixed at a 1∶3 SipS∶RsiV molar ratio. Reactions were incubated for 6 h at 37°C. The role of HEW lysozyme (0.3 mg/mL) as an activator of the RsiV cleavage was also evaluated. The cleavage of RsiV was assessed by the products observed at 26 kDa and 17 kDa and confirmed by Immunoblot analysis.

### Immunoblot Analysis

Strains were grown for 16 hours in LB at 37°C. The cells were subcultured 1∶100 in LB+1 mM IPTG at 37°C and grown to an OD_600_ of 0.8–1. The cells were pelleted by centrifugation and resuspended in 100 µl of 2× Laemmli sample buffer and lysed by repeated sonication. Samples were electrophoresed on a 15% SDS Polyacrylamide gel (BioRad). The proteins were then blotted onto nitrocellulose. The proteins were detected by incubating with a 1∶10,000 dilution of anti-RsiV^59–285^ antibodies [14] or 1∶15,000 dilution of anti-σ^A^ antibodies followed by 3 washes and incubation in a 1∶10,000 dilution of goat anti-rabbit IgG (H+L) IRDye 800CW (Li-Cor) and imaged on an Odyssey CLx (Li-Cor). Quantification of band intensities was performed using Image Studio software (Li-Cor).

### Expression and Purification of R-Lysozyme

*P. pastoris* was grown overnight in 5 ml BMGY (Buffered Glycerol Complex Medium; 100 mM potassium phosphate, pH 6.0, 1% yeast extract, 2% peptone, 1.34% Yeast Nitrogen Base, 0.0004% biotin, 1% glycerol) at 30°C and then subcultured into 300 ml BMGY overnight 30°C in 2L baffled flasks. Cultures were pelleted and washed with BMMY (Buffered Methanol Complex Medium; 100 mM potassium phosphate, pH 6.0, 1% yeast extract, 2% peptone, 1.34% Yeast Nitrogen Base, 0. 0004% biotin, 0.5% methanol) [73]. The cell pellet was resuspended in 300 ml BMMY and grown at 30°C for 5 days. Methanol to a final concentration of 5% was added each day. Cultures were pelleted by centrifugation and the supernatant was clarified by filtering through a 0.2 µm filter. The supernatant was exchanged into 50 mM sodium acetate pH 6.2, and concentrated by running the supernatant over a 3 kDa carbon fiber filter (AG Technologies Corporation). Further purification was performed by FPLC using a Capto S exchange column (GE Healthcare Life Sciences) and a 3 kDa micron filter unit (Millipore). Protein concentration was determined by reading the absorbance at 280 nm.

### Measurement of Lysozyme Activity

Lysozyme activity was measured by the rate of *Micrococcus lysodeikticus* peptidoglycan clearing at 450 nm as previously described [74]. Briefly, *M. lysodeikticus* peptidoglycan was suspended to an OD_600_ of 0.9 and mixed with equal volumes of either buffer alone (50 mM NaOAc pH 6.2), HUM Lysozyme 20 µg/ml, R-lysozyme 20 µg/ml or R-lysozyme^D52S^. The rate of peptidoglycan clearing was monitored at 450 nm for 10 minutes.

### Protoplast with Mutanolysin

Overnight cultures were subcultured 1∶100 in LB+1 mM IPTG and grown to an OD_600_ of 0.8–1. Cells were pelleted by centrifugation, washed with PBS, and resuspended in equal parts 1 M sucrose and 60 mM Tris-Cl, 4 mM MgCl_2_ to form a protoplast buffer [75], [76]. Mutanolysin was added to a final concentration of 2 µg/ml, and incubated shaking at 37°C for 40 minutes. Protoplast were confirmed by phase contrast microscopy. Protoplast samples were left untreated or treated with lysozyme (2 µg/ml) for 10 minutes. An equal volume of 2× Laemmli sample buffer was added to the sample before for immunoblot analysis as described.

### Expression of Recombinant Proteins in *E. coli*

Overnight cultures of *E. coli* BL21λDE3 containing either pKBW201 (pDEST17-*6×his-2×flag*-*rsiV* ^59–285^), pKBW204 (*gst*-*rsiV* ^59–285^), pKBW216 (pDEST17-*6×his*-*rsiV* ^CD^) or pJH227 (pDEST17-*6×his*-*rsiV* ^EF^) were grown at 30°C in LB+ampicillin. The cell cultures were diluted 1∶100 into 500 ml of LB+ampicillin in 2 L baffled flasks and incubated at 30°C to an OD_600_ of 0.5–0.6. IPTG was added to a final concentration of 1 mM to induce protein production and the cultures incubated for an additional 4 hours. Cells were chilled on ice and collected by centrifugation at 5000× g. Cell pellets were stored at −80°C until time for purification.

### Purification of 6×His-Tagged Proteins from *E. coli*

Cell pellets were thawed on ice and resuspended in 5 ml lysis buffer (50 mM Sodium Phosphate, 250 mM NaCl, 10 mM imidazole, pH 8.0) per 500 ml of initial culture volume. Cells were lysed by passaging through a Microfluidics LV1 high shear microfluidizer (Newton, MA) twice. Lysate was centrifuged at 15,000× *g*, for 30 minutes at 4°C, to pellet cellular debris. Cleared lysate was applied to a nickel affinity column to bind 6×His-tagged protein (Qiagen). The column was washed with 10 column volumes of wash buffer (50 mM sodium phosphate, 250 mM NaCl, 20 mM imidazole, pH 8.0). Protein was eluted with elution buffer (50 mM Sodium Phosphate, 250 mM NaCl, 250 mM imidazole, pH 8.0) and collected in 0.5 ml fractions. Samples from each fraction were analyzed by SDS-PAGE and elution fractions containing the desired protein were combined. Combined fractions were then dialyzed into lysis buffer to remove the excess imidazole. The protein was further purified with a GE Healthcare AKTA FPLC (GE Healthcare Sciences Pittsburg, PA) using a HisTrap HP nickel affinity column. Fractions containing the 6×His-2×Flag-RsiV^59–285^ were again combined and dialyzed into a storage buffer (25 mM Tris, 200 mM NaCl, 5% glycerol, pH 8.0) and flash frozen at −80°C until use.

### Purification of GST-Tagged Proteins from *E. coli*

Cell pellets were thawed on ice and resuspended in 2.5 ml PBS-EW (50 mM NaH_2_PO_4_, 150 mM NaCl, 1 mM DTT, 1 mM EDTA, pH 7.2) per 250 ml of initial culture volume. Cells were lysed by 2× passage through a Microfluidics LV1 high shear microfluidizer (Newton, MA). Lysate was centrifuged at 15,000× *g*, for 30 minutes at 4°C, to pellet cellular debris. Cleared lysates were then passed over a Glutathione HiCap Matrix column (Qiagen Valencia, CA). The column was washed with 5 column volumes of PBS-EW. Protein was eluted with buffer TNGT (50 mM Tris, 0.4 M NaCl, 50 mM reduced L-Glutathione, 0.1% Triton-x-100, 1 mM DTT) and collected in 0.5 ml fractions. Samples from each fraction were analyzed by SDS-PAGE and elution fractions containing the desired protein were combined. Purified GST-RsiV^59–285^ was then dialyzed into a buffer containing 50 mM Tris-HCl pH 7.5, 200 mM NaCl and stored at 4°C until use.

### Co-purification Experiments

For 6×His-tagged proteins, expression and purification was performed described above, with the following alterations. After the initial application of wash buffer 1, 2 ml of 1 mg/ml HEW lysozyme (Sigma Aldrich) was applied to the column and flow through collected. An additional 5 column volumes of wash buffer 1 was applied to remove any unbound HEW lysozyme. Elution of the proteins then proceeded as described. Samples from each fraction were mixed with an equal volume with 2× Laemmli sample buffer and analyzed on 15% SDS-PAGE gels stained with Coomassie brilliant blue. To ensure lysozyme binding was not the result of interactions with contaminating proteins from the expression strain or spurious binding to the Ni resin (Thermo-Fisher), mock-expression cells were used in a pull-down experiment as a negative control. Briefly, BL21λDE3 cells containing no plasmid were grown and processed as described. Cleared lysates from these cells were applied to a Ni affinity column in which lysozyme was then passed over. The column was washed and protein eluted under the same conditions as our normal pull-down assay.

Co-purification assays utilizing GST-tagged proteins were completed in a similar manner with the following modifications. Purified GST-tagged protein was dialyzed into PBS-EW and 2 mg of protein was applied to a Glutathione HiCap Matrix column. The column was washed with 5 column volumes of PBS-EW. 2 mg of HEW lysozyme, human lysozyme, or mutanolysin, in PBS-EW, was applied to the column. PBS-EW buffer and TNGT buffer were used as wash and elution buffers, respectively. Samples were analyzed by SDS-PAGE.

### Isothermal Titration Calorimetry

The affinity of the interaction (*K_d_*) between RsiV and HEW lysozyme was determined by isothermal titration calorimetry (ITC). Purified 6×His-2×Flag-RsiV^59–285^ was purified as described above. HEW lysozyme ≥98% pure was purchased from Sigma Aldrich. The proteins were co-dialyzed three times in 2 liters of 50 mM Na_2_HPO_4_, 200 mM NaCl, and pH 7.0 buffer for 8 h (each) at 4°C. Final protein concentrations as determined by absorbance at OD280 were adjusted to 6×His-2×Flag-RsiV^59–285^ (0.01 mM) and HEW lysozyme (0.1 mM) with filtered dialysate. The protein samples were degassed and ITC measurements recorded using a MicroCal VP-ITC System (GE Healthcare) with HEW lysozyme as the injected sample and 6×His-2×Flag-RsiV^59–285^ as the cell sample. 21 injections of HEW lysozyme were used, with 180 seconds spacing between events. The chamber was kept under constant stirring at 350 rpm and all experiments were performed at 25°C. The binding reaction reached saturation during the experiment and control experiments where HEW lysozyme was injected into buffer showed that the heats of dilution were constant across all injections. The constant heat of dilution, as determined by the average of the last 3–5 injections, was subtracted and the data are analyzed using the single site binding model provided in the ITC analysis package. The values for affinity, stoichiometry (n) and change in enthalpy (ΔH) and entropy (ΔS) obtained from three independent experiments were averaged and the standard deviation determined.

## Supporting Information

Figure S1 Purification of RsiV-6×His for N-terminal sequencing. *B. subtilis* strain *amyE*::P*_hs_*-*rsiV-6×his* (JLH548) was grown to an OD_600_ of 1 and then subcultured 1∶100 into 1 L LB supplemented with 1 mM IPTG. Cells were grown to an OD_600_ of 0.8 and then pelleted by centrifugation. The cell pellet was resuspended in protoplast buffer with lysozyme for 45 minutes at 37°C and then centrifuged again. The resulting supernatant was batch purified using Ni resin. Each fraction produced during purification was electrophoresed by SDS PAGE (**A**), and the fraction containing RsiV (elution 2) was confirmed by western blot with anti-RsiV antibodies (**B**). This fraction was transferred to a PVDF membrane, cut out, and sent to Iowa State University for Edman Degradation N-terminal sequencing.
(PDF)

Figure S2 Alignment of *B. subtilis*, *E. faecalis*, *C. difficile* RsiV. **A.** Alignment of *B. subtilis*, *C. difficile* and *E. faecalis* RsiV. In bold is the putative transmembrane domain as predicted by TMHMM [18]; highlighted in yellow is a consensus sequence identified by MEME [17] in Figure S2B and in red is the putative signal peptidase recognition site and the cleavage sites as denoted by a space as predicted by SignalP 4.1 Server [16]. **B.** Alignment was generated by MEME [17] using 185 homologs of RsiV from NCBI as of March 25, 2014.
(PDF)

Figure S3 Δ*sipS::cat* and Δ*sipT::tet* do not block RsiV degradation. *B. subtilis* strains Δ*sigVrsiV::kan* P*_hs_*-*rsiV^+^* (JLH402), Δ*sigVrsiV::kan* P*_hs_*-*rsiV* Δ*sipS::cat* (JLH933) and Δ*sigVrsiV::kan* P*_hs_*-*rsiV* Δ*sipT::tet* (JLH953) were grown in LB+1 mM IPTG to an OD of 0.8. Samples were either treated with 2 µg/ml lysozyme (+) or untreated (−) and incubated for 10 minutes 37°C. Immunoblot was probed with anti-RsiV^59–285^ antibodies.
(PDF)

Figure S4 *In vitro* transcription translation of RsiV and signal peptidase SipS. Coomassie gel showing the partially purified cell free production of RsiV and SipS used for *in vitro* cleavage assays. GFP was used as a production control. Lane 1 Ladder; Lane 2 GFP pellet (P); Lane 3 GFP supernatant (S); Lane 4 RsiV pellet; Lane 5 RsiV supernatant; Lane 6 RsiV^A66W^ pellet; Lane 7 RsiV^A66W^ supernatant; Lane 8 SipS pellet; Lane 9 Sip supernatant. Arrows denote location of proteins on the gel FL-RsiV (3×Fag-CBP-RsiV-6×His) and SipS.
(PDF)

Figure S5 Purified RsiV (RsiV^59–285^) and HEW Lysozyme used in ITC experiments. Recombinant 6×His-2×FLAG-RsiV^59–285^ was purified as described and dialyzed into 50 mM Na_2_HPO_4_, 200 mM NaCl, pH 7.0. HEW Lysozyme, ≥98% pure (Sigma Aldrich), was reconstituted in the same buffer and co-dialyzed with the RsiV protein. Samples of each were subjected to SDS-PAGE on 15% gels and stained with coomassie.
(PDF)

Figure S6 Activity of R-lysozyme and R-lysozyme^D53S^. Activity of Recombinant lysozyme (R-lysozyme) and R-lysozyme^D52S^ was assayed by mixing *M. lysodekticus* (OD_600_ = 0.9) was mixed equally with buffer (50 mM NaAc pH 6.2), human lysozyme (20 µg/ml), R-lysozyme (20 µg/ml), or R-lysozyme^D53S^ (20 µg/ml) and the OD450 was measured every minute for 10 minutes to monitor *M. lysodeikticus* peptidoglycan degradation. As expected the R-lysozyme was active while the R-lysozyme^D52S^ was inactive.
(PDF)

Table S1 Oligonucleotide primers.
(DOCX)
