# Supplementary figures and images for: Evidence of a Bacterial Receptor for Lysozyme: Binding of Lysozyme to the Anti-σ Factor RsiV Controls Activation of the ECF σ Factor σV
Source: PLoS Genet. 2014 Oct 2;10(10):e1004643. doi: 10.1371/journal.pgen.1004643 (PMC4183432; doi:10.1371/journal.pgen.1004643)

**A**

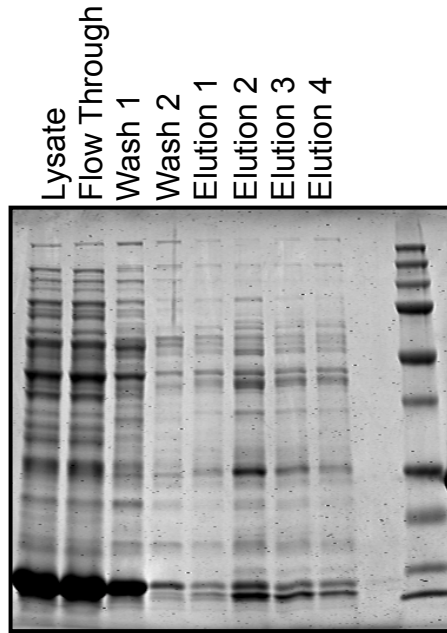

**B**

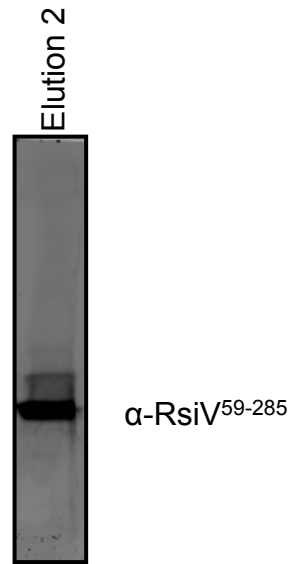

Supplement: Figure S1 — Purification of RsiV-6×His for N-terminal sequencing. B. subtilis strain amyE::Phs-rsiV-6×his (JLH548) was grown to an OD600 of 1 and then subcultured 1∶100 into 1 L LB supplemented with 1 mM IPTG. Cells were grown to an OD600 of 0.8 and then pelleted by centrifugation. The cell pellet was resuspended in protoplast buffer with lysozyme for 45 minutes at 37°C and then centrifuged again. The resulting supernatant was batch purified using Ni resin. Each fraction produced during purification was electrophoresed by SDS PAGE (A), and the fraction containing RsiV (elution 2) was confirmed by western blot with anti-RsiV antibodies (B). This fraction was transferred to a PVDF membrane, cut out, and sent to Iowa State University for Edman Degradation N-terminal sequencing. (PDF) [file pgen.1004643.s001.pdf]

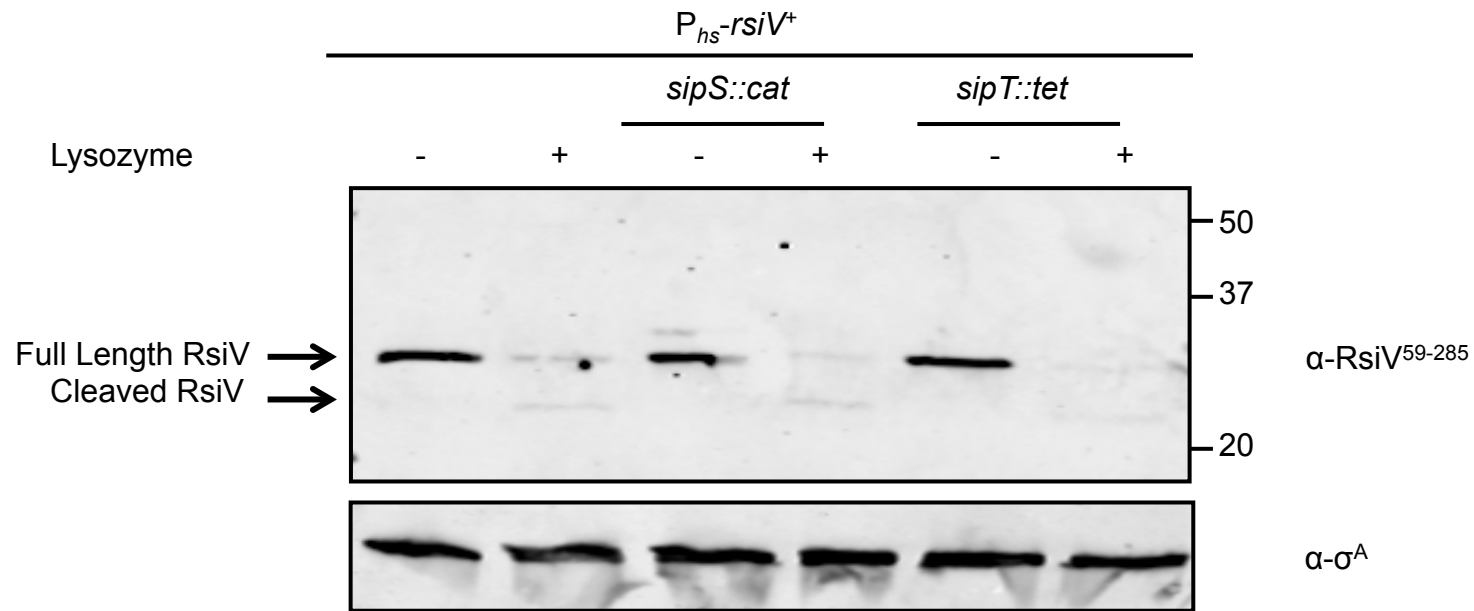

Supplement: Figure S3 — ΔsipS::cat and ΔsipT::tet do not block RsiV degradation. B. subtilis strains ΔsigVrsiV::kan Phs-rsiV+ (JLH402), ΔsigVrsiV::kan Phs-rsiV ΔsipS::cat (JLH933) and ΔsigVrsiV::kan Phs-rsiV ΔsipT::tet (JLH953) were grown in LB+1 mM IPTG to an OD of 0.8. Samples were either treated with 2 µg/ml lysozyme (+) or untreated (−) and incubated for 10 minutes 37°C. Immunoblot was probed with anti-RsiV59–285 antibodies. (PDF) [file pgen.1004643.s003.pdf]

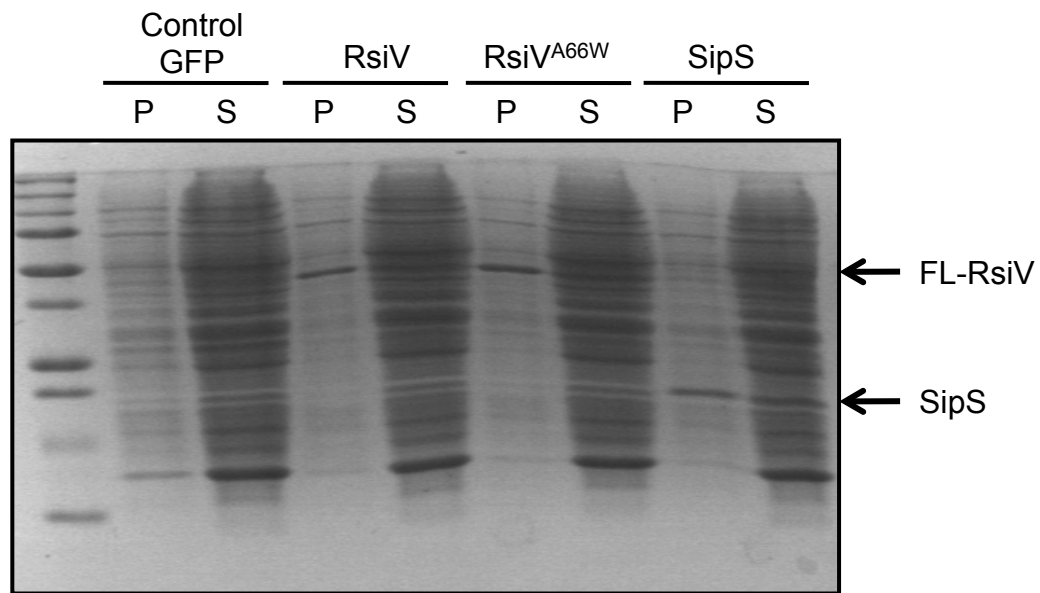

Supplement: Figure S4 — In vitro transcription translation of RsiV and signal peptidase SipS. Coomassie gel showing the partially purified cell free production of RsiV and SipS used for in vitro cleavage assays. GFP was used as a production control. Lane 1 Ladder; Lane 2 GFP pellet (P); Lane 3 GFP supernatant (S); Lane 4 RsiV pellet; Lane 5 RsiV supernatant; Lane 6 RsiVA66W pellet; Lane 7 RsiVA66W supernatant; Lane 8 SipS pellet; Lane 9 Sip supernatant. Arrows denote location of proteins on the gel FL-RsiV (3×Fag-CBP-RsiV-6×His) and SipS. (PDF) [file pgen.1004643.s004.pdf]

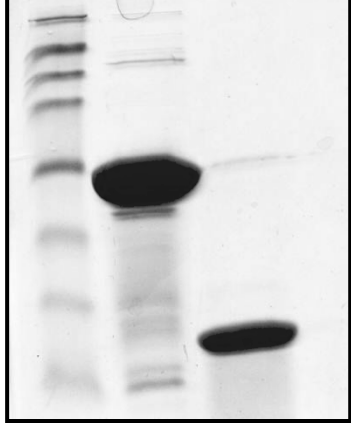

6xHis-RsiV<sup>59-285</sup>

HEW Lysozyme

Supplement: Figure S5 — Purified RsiV (RsiV59–285) and HEW Lysozyme used in ITC experiments. Recombinant 6×His-2×FLAG-RsiV59–285 was purified as described and dialyzed into 50 mM Na2HPO4, 200 mM NaCl, pH 7.0. HEW Lysozyme, ≥98% pure (Sigma Aldrich), was reconstituted in the same buffer and co-dialyzed with the RsiV protein. Samples of each were subjected to SDS-PAGE on 15% gels and stained with coomassie. (PDF) [file pgen.1004643.s005.pdf]

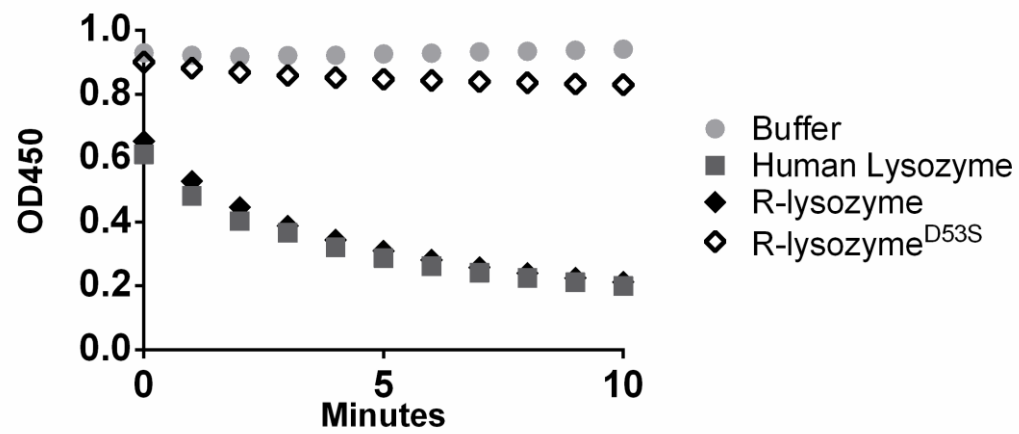

Supplement: Figure S6 — Activity of R-lysozyme and R-lysozymeD53S. Activity of Recombinant lysozyme (R-lysozyme) and R-lysozymeD52S was assayed by mixing M. lysodekticus (OD600 = 0.9) was mixed equally with buffer (50 mM NaAc pH 6.2), human lysozyme (20 µg/ml), R-lysozyme (20 µg/ml), or R-lysozymeD53S (20 µg/ml) and the OD450 was measured every minute for 10 minutes to monitor M. lysodeikticus peptidoglycan degradation. As expected the R-lysozyme was active while the R-lysozymeD52S was inactive. (PDF) [file pgen.1004643.s006.pdf]
