# Supplementary material for: Evidence of a Bacterial Receptor for Lysozyme: Binding of Lysozyme to the Anti-σ Factor RsiV Controls Activation of the ECF σ Factor σV
Source: PLoS Genet. 2014 Oct 2;10(10):e1004643. doi: 10.1371/journal.pgen.1004643 (PMC4183432; doi:10.1371/journal.pgen.1004643)

A.

```
B. subtilis RsiV    ----MDKRLQQLREEYKNVQIPKELDIIVEKALQQ---EPKKKRIVMWPT 43
C. difficile RsiV   --MTSKERLNKLKDEYHKIPIPKKLDTIINHEKIENIYREKNKKVNRLRF 48
E. faecalis RsiV    MEDFVKSVVKNFSKEYRQQPLPTDLQAEVKARFHKEKKRYTWLRFRNRSL 50
                          .. :::: .**::  :*..*:  ::    :   . .  :.

B. subtilis RsiV    SAAIAAAILFTALVNINPDAAQA MSKIPVIGKIVKAITFIEIKEEKDQSS 93
C. difficile RsiV   KVAIAFACIFTVLVNISPVFA DNFSKIPVIGAIVEVITIKNYSLKSENYE 98
E. faecalis RsiV    QSSLVVACGFVLSVNLFPGFSEAA RNIPVLDKIVQLVTIKTLTAKKQESE 100
                    . ::. *  *.  **: *       :***:. **: :*:   . :.:: .

B. subtilis RsiV    IDVKTPALSGLSNKELENSINEKYLKESQQLYKEFIQSTSKNK----KGH 139
C. difficile RsiV   AEIDIPKIRGLKDKNLEQRLNSSFMEDGKRLYHQFQERMEKIQSSKNKGY 148
E. faecalis RsiV    VNIDVPKIQTSQESSVADTLNKKYLKEAQTEFQQVKGQLFDGS------R 144
                    ::. * :   .:..: : :*..:::::.: :::.     . .

B. subtilis RsiV    LSIYSDYETVTDTPDLLSIRRNIETTQASSYTQSRYITIDKKNDILLTLK 189
C. difficile RsiV   KSLSLSYSVKNNSKKFLSIEMTKNEIEASSYVSKVHYTIDKKRQIVLTLP 198
E. faecalis RsiV    VSVTGDYEKVVDDRRFLVVKRTFTEIKGSSATTTKYDTIDKRANVVVSLP 194
                     *:  .*.   :   :* :. .    :.** . . : ****: :::::*

B. subtilis RsiV    SLFKDERYIKVISQNIKEQMKQQMKEDPNKIYWLT----DEDAEPFKTIL 235
C. difficile RsiV   MLFKDDKYIKVISDNIKEQMREQMKKDSTKSYFIDQK-KDLPVEDFKTIN 247
E. faecalis RsiV    LIFKNDFYIDVISSEIKQQISDQMKNDSNKIYWSEKEDAPSDVQPFKKIK 244
                     :**:: **.***.:**:*: :***:*..* *:         .: **.*

B. subtilis RsiV    PDQTFYITEDHKLVISFDEYEVAPGYMGVTEFTIPTGVISNLLVGERYIR 285
C. difficile RsiV   KYQDFYFNKNEDLVICFDEYEVAPGYMGAVEFVIPYKVIKDL-------- 289
E. faecalis RsiV    KDQPFYINEKHQLVIVFPQGEIAPYYMGTPEFVIPNQVIENELAAPNYLK 294
                      * **:.:...*** * : *:** ***. **.**  **.:
```

B.

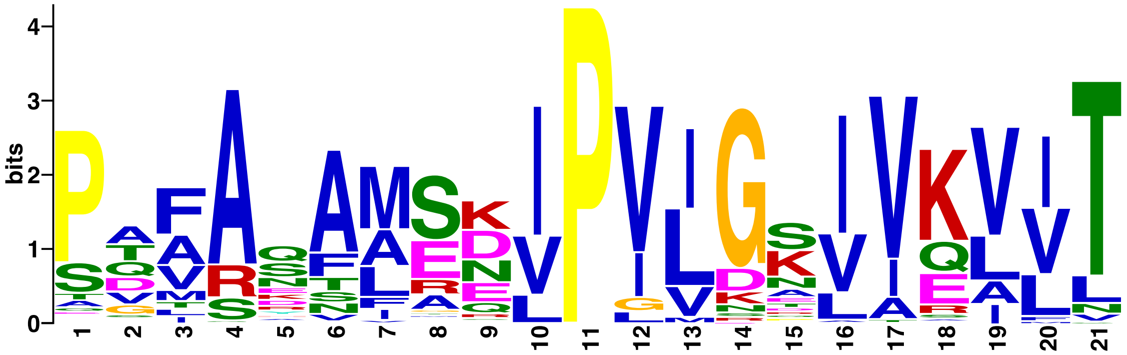

Supplement: Figure S2 — Alignment of B. subtilis, E. faecalis, C. difficile RsiV. A. Alignment of B. subtilis, C. difficile and E. faecalis RsiV. In bold is the putative transmembrane domain as predicted by TMHMM [18]; highlighted in yellow is a consensus sequence identified by MEME [17] in Figure S2B and in red is the putative signal peptidase recognition site and the cleavage sites as denoted by a space as predicted by SignalP 4.1 Server [16]. B. Alignment was generated by MEME [17] using 185 homologs of RsiV from NCBI as of March 25, 2014. (PDF) [file pgen.1004643.s002.pdf]
