# Supplementary material for: Evidence of a Bacterial Receptor for Lysozyme: Binding of Lysozyme to the Anti-σ Factor RsiV Controls Activation of the ECF σ Factor σV
Source: PLoS Genet. 2014 Oct 2;10(10):e1004643. doi: 10.1371/journal.pgen.1004643 (PMC4183432; doi:10.1371/journal.pgen.1004643)
Supplement: Table S1 — Oligonucleotide primers. (DOCX) [file pgen.1004643.s007.docx]

**Table S1 Oligonucleotide Primers**

| **Primer** | **Sequence 5’ to 3’** | **Description of use** |
| --- | --- | --- |
| CDEP1544 | gtttttattttaatggtgatggtgatggtgtcgaatataacgttc | Clone IPTG-inducible *rsiV-6xhis* |
| CDEP1430 | cacctaaggaggctttcttaatggataagagattacagc | Clone IPTG-inducible *rsiV-6xhis* |
| CDEP1561 | atcaacccggacgccgctcagtggatgtcaaagatccct | Clone *rsiV^A66W^* |
| CDEP1562 | agggatctttgacatccactgagcggcgtccgggttgat | Clone *rsiV^A66W^* |
| CDEP1610 | aaagcatgcggatccgtttaaacgagctcaccctgaaaatac | Clone *3xflag-cbp* |
| CDEP1611 | gatcatgatatcgactacaaagacgatgacgacaagcacgtgtccatggaaaagagaag | Clone *3xflag-cbp* |
| CDEP1612 | aaaagctttaaggaggatttatagatggattataaggatcatgatggtgattataaggatcatgatatcgactac | Clone *3xflag-cbp* |
| CDEP1140 | caccatggactacaaagacgatgacgacaaggactacaaag | Clone *6xhis-2xflag--rsiV* |
| CDEP952 | ccataatcttagatggatg | Clone *6xhis-2xflag--rsiV* |
| CDEP950 | cacctaaggaggatttatagatggactacaaagacgatgacgacaaggactac | Clone *6xhis-2xflag--rsiV* |
| CDEP1678 | cacctaaggagggcgatcgcatgaaatcagaaaatgtttcgaag | Clone *sipS* |
| CDEP1679 | aagagctcgctgcttgatcctaatttgttttgcg | Clone *sipS* |
| CDEP1677 | aagcgatcgcatggattataaggatcatgatggtg | Clone *3xflag-cbp-rsiV-6xhis* |
| CDEP1714 | aaacccgggttaatggtgatggtgatggtgtcgaatataacgttctcccacg | Clone *3xflag-cbp-rsiV-6xhis* |
| CDEP1954 | ctcactaaagggaacaaaagctggagtacagtcggcattatctc | *cat* universal PCR primers |
| CDEP1955 | acgactcactatagggcgaattgtccttacgcgaaatacgggc | *cat* universal PCR primers |
| CDEP1697 | tttattcctcatcgggctgagccgt | Δ*sipS::cat* |
| CDEP1709 | caattcgccctatagtgagtcgtatcaacgacatacggcgcaa | Δ*sipS::cat* |
| CDEP1710 | ccagcttttgttccctttagtgagacggtcatcccctaaaaacagaaca | Δ*sipS::cat* |
| CDEP1700 | ttacgacaacagcgccgacg | Δ*sipS::cat* |
| CDEP1701 | ttacgacaacagcgccgacg | Δ*sipT::tet* |
| CDEP1711 | caattcgccctatagtgagtcgtaaagtgacggatcaggagagcca | Δ*sipT::tet* |
| CDEP1712 | ccagcttttgttccctttagtgagtgctggcctaggacagcagg | Δ*sipT::tet* |
| CDEP1704 | ggtggttcgtcggctcatcga | Δ*sipT::tet* |
| CDEP1892 | catgccatggtacccgggattcaaaaaggagccctttattg | *rsiV^A66W^* for chromosomal mutant |
| CDEP1893 | tagaattcgagctcccgggccataatcttagatggatgtttttattttatc | *rsiV^A66W^* for chromosomal mutant |
| CDEP1434 | caccgaagcagcccgaaacattc | Clone *E. faecalis rsiV* |
| CDEP1435 | ttattttaaataattaggcg | Clone *E. faecalis rsiV* |
| CDEP928 | caccatggcagacaatttttctaag | Clone *C. difficile* *rsiV* |
| CDEP189 | gtagtcatattgtcctc | Clone *C. difficile* *rsiV* |
| CDEP1888 | ctaaagaagaaggggtatctctcgagaaaagaaaggtctttgaaagatgtgag | Clone *hlyz* |
| CDEP1889 | ctgagatgagtttttgttcgcgaattcttattaaacacc | Clone *hlyz* |
| CDEP1847 | ggtgacagatctacttcttatggtatatttcaaatc | Clone *hlyz^D53S^* |
| CDEP1848 | gatttgaaatataccataagaagtagatctgtcacc | Clone *hlyz^D53S^* |
